# New diagnostic criteria for headache attributed to transient ischemic attacks

**DOI:** 10.1186/s10194-019-1041-9

**Published:** 2019-09-06

**Authors:** Elena R. Lebedeva, Natalia M. Gurary, Jes Olesen

**Affiliations:** 1 Department of Neurology, the Ural State Medical University, Repina 3, Yekaterinburg, 620028 Russia; 2 International Headache Center “Europe-Asia”, Yekaterinburg, Russia; 3 Medical Union “New Hospital”, Yekaterinburg, Russia; 4 Danish Headache Center, Department of Neurology, Rigshospitalet-Glostrup, University of Copenhagen, Copenhagen, Denmark

**Keywords:** Transient ischemic attack, ТIA, Headache attributed to transient ischemic attacks, Headache in TIA, Diagnostic criteria

## Abstract

**Background:**

The International Classification of Headache Disorders diagnostic criteria for Headache Attributed to Transient Ischemic Attack (TIA) and many other secondary headaches are based primarily on the opinion of experts. The aim of this study was to field test, for the first time, the diagnostic criteria for headache attributed to TIA of the International Classification of Headache Disorders, 3rd edition (ICHD-3) and in case of their weaknesses to propose new diagnostic criteria.

**Methods:**

Consecutive patients with Transient Ischemic Attack and a simultaneous control group were extensively interviewed soon after admission. Data were collected on previous headaches, headaches around the time of Transient Ischemic Attack and characteristics of the TIA using validated neurologist conducted semi-structured interview forms. The evidence of relevant infarction were excluded in patients with Transient Ischemic Attack using magnetic resonance imaging with diffusion-weighted imaging (*n* = 112) or *computed tomography* (*n* = 8).

**Results:**

One hundred twenty patients with Transient Ischemic Attack and 192 controls were included. A new type of headache occurred within 24 h in 16 (13%) of patients with Transient Ischemic Attack and in no controls, a preexisting type of headache with altered characteristics occurred in 9 (7.5%) of patients with Transient Ischemic Attack and no in controls, headache without altered characteristics occurred in 8 (6.6%) of patients with Transient Ischemic Attack and in 9 (4.6%) controls. Only 24% of the headaches in patients with Transient Ischemic Attack (8 of 33 patients) fulfilled the diagnostic criteria of International Classification of Headache Disorders-3 and no control patients. We propose new criteria fulfilled by 94% of the headaches. Specificity remained excellent as only one of 192 controls had a headache fulfilling the proposed criterion C.

**Conclusions:**

Existing diagnostic criteria for headache attributed to TIA of the International Classification of Headache Disorders are too insensitive. We suggest new diagnostic criteria with high sensitivity and preserved specificity.

## Introduction

At the time of development and publication of the International Classification of headache Disorders 3rd edition (ICHD-3) no studies had provided exact information about timing and clinical characteristics of the headaches attributed to transient ischemic attack (TIA) and the headaches had not been compared to a thorough diagnosis of patients’ previous headache disorders [1–8]. Therefore, existing diagnostic criteria for headache attributed to TIA (ICHD-3) are based primarily on the opinion of experts rather than on published clinical evidence [9].

Recently we performed an extensive prospective study of 120 consecutive patients with TIA [10]. As part of this study we performed extensive semi-structured interviews about previous and actual headaches as soon after admission for TIA as possible. Thus, we characterised both the previous headaches and the headaches occurring around the time of TIA. This has allowed us to evaluate which types of headaches that occur, exactly when they occur and how long they last. For each type of headache associated with TIA we furthermore compared to a simultaneous control material investigated in the same way. The aim of this study was to field test, for the first time, the diagnostic criteria for headache attributed to TIA of the International Classification of Headache Disorders, 3rd edition (ICHD-3) and in case of their weaknesses to propose new diagnostic criteria.

## Material and methods

The period of recruitment of patients was from April 2014 till May 2016. We previously described material and methods [10]. Here we bring an abbreviated version. Patients were eligible for enrollment if they had had a TIA and were admitted to the stroke unit of city hospital “New Hospital” in Yekaterinburg, Russia. Eligible patients were diagnosed according to existing definition of TIA [11] and had focal brain or retinal ischemia with resolution of symptoms within 24 h without presence of new infarction on magnetic resonance imaging with diffusion-weighted imaging (*n* = 112) or *computed tomography* (*n* = 8) which were done at the time of admission to the hospital. After that all patients were evaluated within one day of admission, usually within a few hours by one neurologist (NMG) who collected all patient data prospectively, using a standardized case-report form during face-to-face interviews. Participants should be able to give a clear description of past and present headaches and agree to conduct interviews and additional telephone interviews in case clarifying questions were needed. Traumatic injury of the head was exclusion criteria in this study as well as other serious neurological or somatic disorders.

A total of 131 patients were examined, 11 patients were excluded because most of them had difficulties to recall essential information and memory problems. One hundred twenty patients with TIA were included in the study. All TIA cases were subdivided into anterior and posterior circulation TIA.

As a control group we used patients who were admitted to the emergency room without acute neurological deficits or serious neurological or somatic disorders. We examined 225 controls. Thirty three patients were excluded and 192 patients were included.

The history of headache in both groups was recorded using extensive semi-structured interview forms that contained all necessary information to diagnose previous and present headaches.

TIA was defined as a *transient episode* of *neurological dysfunction caused* by *focal brain, spinal cord,* or *retinal ischemia, without acute infarction* [11]. The diagnoses of previous and present headaches were made according to the explicit diagnostic criteria of the ICHD-3 [9]. We used this classification for testing of the criteria for Headache Attributed to TIA. We recorded headache within the last year and within 24 h after onset of TIA or within 24 h after admission of controls. We distinguished between previous headache without change of characteristics, headache with change of characteristics and new type of headache. We defined a new type of headache in TIA as a headache which arose for the first time within 24 h after onset of TIA. We indicated types and time of headache development in TIA and time of disappearance of headache. To make clear differential diagnosis between TIA and migraine with aura we included questions about gradual spread or sudden development of symptoms, presence of positive and negative symptoms and information how all symptoms occur: simultaneously or not [12].

### Ethical considerations

The Medical Ethics Committee of the Urals State Medical University approved this study. All respondents were informed of the purpose of the study. Written informed consent was obtained from all participants.

### Statistical analysis

All 120 patients with TIA included in this study were submitted to testing of the criteria for Headache Attributed to TIA. The number of patients fulfilling each criterion was recorded. Sensitivity was calculated as a number fulfilling a set of criteria over all headaches of the same category.

## Results

General characteristics of the material have been published before [10]. The mean age of patients with TIA and controls did not differ significantly: 56.1 and 58.7 respectively but there females were overrepresented in the control group. Control patients had the following diagnoses: “lumbago” or “lumbar spine osteochondrosis” (*n* = 99), “pancreatitis” (n= 62), “gastrointestinal ulcer” (*n* = 7), tick bite (*n* = 14), irritable bowel syndrome (*n* = 2), paroxysmal benign positional vertigo (*n* = 2), arthritis (*n* = 5), allergic reaction (*n* = 1). Most patients (106 patients, 88%) had TIA in the anterior circulation system and only few (14 patients, 12%) in the posterior circulation system. The duration of TIA varied from 5 min to 24 h. Forty two patients (35%) had resolution of all symptoms within 60 min. Seven patients (5.8%) had two or more attacks of TIA.

**Fig. 1:**
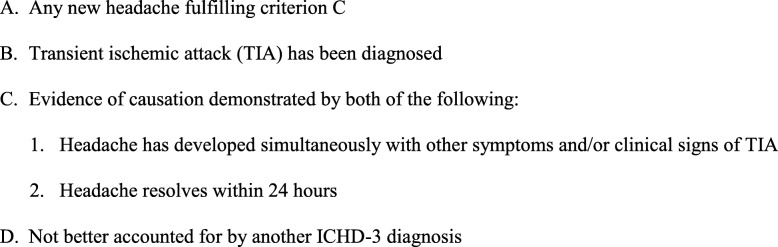
Headache Attributed to TIA according to ICHD-3

The existing diagnostic criteria for Headache Attributed to TIA of the ICHD-3 are presented in Fig. 1. We were in doubt about C1 criteria: “Headache has developed simultaneously with other symptoms and/or clinical signs of TIA” and C2 criteria: “Headache resolves within 24 hours”. For field testing of diagnostic criteria we evaluated all previous and present headaches in patients with TIA. Of 120 consecutive patients with TIA, 75% already had previously tension-type headache (TTH) and 22.5% had migraine during previous year [10]. We paid a special attention to headaches which arose at the time of TIA and 24 h after TIA onset because they can be caused by TIA. We subdivided all headache into new type of headache and previous headaches with and without changes in clinical characteristics. Besides we evaluated their duration.

### Types and time of headache development in TIA

A new type of headache occurred in 16patients (13%) within 24 h after TIA onset (Table 1). Of these, 12 occurred simultaneously (defined as within 1 h of TIA onset) with TIA and 4 occurred 2–24 h after TIA onset. No controls had a new type of headache.

**Table 1:** Fulfilment of ICHD-3 diagnostic subcriteria and proposed subcriteria for headache attributed to TIA

Diagnostic criteria	Fulfilment of diagnostic criteria (number of patients with TIA)
A. ICHD-3 diagnostic criteria	
New kind of headache	16
Onset not simultaneous with TIA	4
Lasting more than 24 h	4
Total fulfilling ICHD-3 criteria	8
B. Proposed diagnostic criteria	
New kind of headache	16
Previous headache with altered characteristics simultaneous with TIA	8
Previous headache without altered characteristics simultaneous with TIA	7
Total fulfilling proposed criteria	31

Headache of a previous type but with changes in clinical characteristics was present in 9 patients (7.5%) and in no controls. These patients included 7 patients with two or more attacks of TIA. A previous type of headache without any change in clinical characteristics was seen in 8 patients (6.6%). Totally 17 patients had headache with and without changes in clinical features. Of these 17 patients 15 had the onset of headache simultaneously with TIA (defined as within 1 h) and 2 started 2–24 h after TIA onset. Headache without changes in clinical features was seen in 9 patients of controls during admission (4.6%).

Thus, we found that a new type of headache and a previous headache with altered characteristics, none of which were seen in the control group, can occur hours after TIA. Therefore criterion C1 of the ICHD-3 is too strict.

### Time of disappearance of headaches in TIA

New type of headaches which developed simultaneously with TIA lasted on average 20.3 h in 12 patients and in 4 lasted more than 24 h (Table 2). The mean duration of headaches with and without changes of characteristics which developed simultaneously with TIA was 27 h in 15 patients and in 5 patients they lasted more than 24 h. These data suggest that headache simultaneously with TIA does not always resolve within 24 h. Therefore criterion C2 for headache attributed to TIA is too strict.

**Table 2:** Sensitivity calculations for ICHD-3 diagnostic criteria and proposed diagnostic criteria

Diagnostic criteria	Number of TIA patients	Sensitivity
A. ICHD-3 diagnostic criteria		
New simultaneous headache lasting ≤24 h over all new headaches occurring within 24 h	8/16	50%
Using all headaches occurring within 24 h as denominator	8/33	24%
B. Proposed diagnostic criteria		
New headaches within 24 h over all headaches within 24 h	16/33	48%
New and simultaneous previous with altered characteristics over all headaches within 24 h	24/33	72%
New plus any simultaneous previous over all headaches within 24 h	31/33	94%

**Fig. 2:**
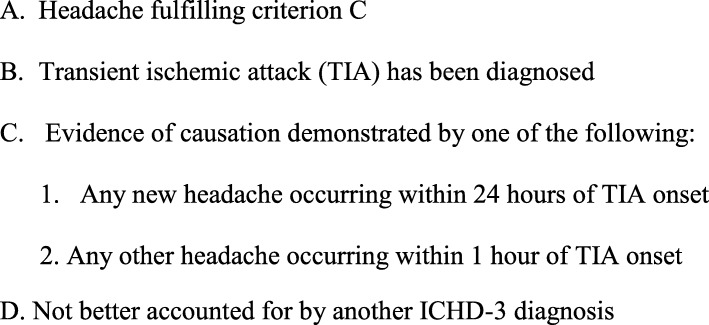
New proposed diagnostic criteria for Headache Attributed to TIA

Totally 33 patients had onset of any kind of headache within one hour of TIA and of a new kind of headache 2–24 h after TIA onset. Applying existing diagnostic criteria for TIA (Table 2) to our material, we found that only 8 of 33 patients fulfilled criteria C1 and C2, resulting in a sensitivity of 24%. Headache of this kind was only seen in one of 192 control patients. According to our data the main limiting factors for the ICHD-3 criteria were the requirement for a new kind of headache, for headache onset at the exact time of TIA onset and for headache to disappear within 24 h (Table 2). Therefore C1 and C2 criteria for headache attributed to TIA need to be modified. We propose new diagnostic criteria (Fig. 2): C1 “Any new headache occurring within 24 hours of TIA onset” and C2 “Any other headache occurring within 1 hour of TIA onset”. We found that 8 of 120 patients fulfilled the existing criteria and 33 of 120 fulfilled the proposed criteria. That have led to significantly greater sensitivity (*p* < 0.0001). The proposed new diagnostic criteria were fulfilled by 0.5% of our controls and no controls fulfilled the existing criteria (*p* = 0.32).

## Discussion

We here present the first prospective study of the ICHD-3 diagnostic criteria for Headache Attributed to Transient Ischemic Attacks (TIA). We propose revised criteria with a much better sensitivity and preserved specificity.

### Development of modified diagnostic criteria for TIA

Before developing new criteria a crucial question had to be considered: which headaches do we really believe are caused by the emboli of a transient ischemic attack? It seems intuitively obvious that a new kind of headache never before encountered by the patient is highly likely to be caused by the TIA, also when it starts some time after the neurological deficits. We decided that all new headaches starting within 24 h after TIA should be included. The likelihood of such headaches starting for other reasons would be infinitesimally small and they were seen in only 1 of 192 control patients. Another issue was headaches of a type previously encountered. The major problem here, which is also relevant for other secondary headaches, is that tension-type headache occurs in 75% of all normal persons. This was also the case in our material of TIA patients. Furthermore, tension-type headache is rather featureless. It means that most secondary headaches cannot be distinguished from TTH on the basis of their clinical characteristics alone. For this reason we decided to include headaches of a previous type that occurred simultaneously with the neurological deficits. No such headaches were seen in 192 control patients underlining the validity of this decision.

What needed modification was, therefore, the requirement under criterion C which establishes causality. The first sub-criterion under C in ICHD-3 says that headache of a new kind must develop simultaneously with TIA. It is unclear what this means. Should headaches developing 5, 30 or 60 min after onset of the neurological deficits be included? We define simultaneous onset as within one hour of the onset of neurological deficits. We also include not only new headaches but any headache occurring within one hour of TIA onset according to the discussion above. The other sub criterion requests that headache should resolve within 24 h. It is unclear why. In our material they often lasted longer than 24 h. Furthermore, secondary headaches due to almost any other cause have no criterion requesting remission of headache within a specified time. That requirement would make it impossible to study if some of these headaches perhaps became long lasting. Our data are the first to show that some headaches in TIA patients persist beyond 24 h. Based on our data and the above reasoning, we propose the criteria presented in Fig. 2.

Using these criteria all patients with any type of headache occurring within one hour and all new headaches occurring within 2–24 h of TIA fulfilled the criteria. The sensitivity of the new criteria was significantly higher than of existing criteria, 94% versus of 24%. The reason for including some patients with headache of a usual type is that the likelihood of a coincidental headache within one hour of TIA onset is minimal even in patients with a pre-existing type of headache as demonstrated in our control material. In fact this could mainly happen in patients with previous chronic headache but only 4% of our patients with TIA had a chronic headache [10]. Likewise, it would be extremely unlikely that a new type of headache might occur by chance on a particular day. This was clearly shown in our control material. Finally, the fact that most of these headaches happened in patients with posterior circulation TIA suggests causality because only 12% of our TIA patients had posterior circulation TIA. Our suggested diagnostic criteria have massively increased sensitivity and preserved specificity because only one of 192 control patients had a headache fulfilling the proposed criteria. Thus, we are confident that the suggested diagnostic criteria represent a significant improvement over the published ICHD-3 criteria for headache attributed to TIA.

### Implications for diagnostic criteria of other secondary headaches

The problem with the diagnostic criteria for all secondary headaches is criterion C which establishes causality. For a few entities the headache has distinct features i.e. headache attributed to low cerebral spinal fluid pressure and thunderclap headache. But for the majority of secondary headaches the causality depends on the temporal association. Previously, in ICHD-1 and ICHD-2, it was easier because it was required that the headache should improve or disappear after treatment of the secondary cause, but that made it impossible to diagnose at the first patient encounter and sometimes impossible all together because the secondary cause could not be treated. ICHD-3 allows diagnosis at the first patient encounter which is a big step forward but at the same time is weaker regarding causality. In the general diagnostic criteria for secondary headaches of ICHD-3, the first sub-criterion under C requires that the headache has developed in temporal relation to the onset of the presumed causal disorder. Because TTH is so extremely prevalent and featureless, many headaches that are caused by another disease or trauma have the characteristics of TTH, which may have existed before the secondary cause of headache. We excluded in the present study possibility of development of headache because of traumatic injury of the head**,** it was exclusion criteria as well as other serious neurological and somatic disorders. Development of headaches connected with medication overuse was also excluded because these headaches developed only in two patients (1.7%) and nobody of them had new type of headache or headache with changes of characteristics. Besides we did not observe alcohol-induced headache in our patients. Only 4 people (3%) among 120 patients used strong alcohol beverages before TIA and they had no headache during TIA.

In the present article we suggest a new principle for headache attributed to TIA that may also be applicable to some other secondary headaches, namely to distinguish between a new kind of headache and a headache of a previous type subdivided into those with usual symptomatology and those with altered symptomatology. For headaches of a new type the temporal association can be less tight than for headaches of a usual type. For the latter we found it unnecessary in TIA with always acute onset to distinguish between those with and those without altered symptomatology. That is, however, different in other secondary headaches. Headache attributed to brain tumor has, for example, often the characteristics of TTH and most patients with a brain tumor already have had TTH. Thus, altered characteristics may be required to diagnose headache attributed to brain tumor. It could be a change in location, intensity or frequency of headaches or occurrence of additional features. But that can only be determined after a thorough analysis of the previous headaches.

### Strengths and weaknesses of the present study

This field testing study capitalizes on extensive prospective data collection using a semi-structured format. Headache attributed to TIA is important because it is common in the emergency room. The study also has some implication for the diagnostic criteria for other secondary headaches. Thus it illustrates how important it is to distinguish between a new type of headache, a usual type with- and without altered characteristics. A significant weakness is that the diagnostic criteria are only tested in one group of TIA patients. They should be tested also in other studies and in other centers.

## Conclusions

We present prospective data demonstrating that existing diagnostic criteria for Headache Attributed to TIA are too insensitive. We have developed new criteria that are highly sensitive and probably not less specific, although the latter needs more study. This way of field testing criteria may be relevant also for other secondary headaches.

### Clinical implications

Current diagnostic criteria for Headache Attributed to TIA have 24% sensitivity.

Proposed new diagnostic criteria for Headache Attributed to TIA have 94% sensitivity.

Our results indicate that the diagnostic criteria for Headache Attributed to TIA should be changed.

## Data Availability

The datasets used and analyzed during the current study are available from the corresponding author on reasonable request.

## Abbreviations

ICHD-3: International Classification of Headache Disorders
TIA: Transient ischemic attack
TTH: Tension-type headache
